# Safety and Efficacy of Busulphan Based on Dosing Patterns in the Real‐World Management of Myeloproliferative Neoplasms

**DOI:** 10.1002/jha2.1097

**Published:** 2025-03-19

**Authors:** Ali Mahdi, Alexandros Rampotas, Patrick Roberts, Joanna Stokes, Eamon Mahdi, Ruth Witherall, Deepak Mannari, Naheed Ibrahim, Georgina Naylor, Mamta Garg, Imran Manjra, Paula Glancy, George Katis, Sahil Bhagat, Jason Coppell, Andrew McGregor, Rebecca Frewin, Nauman M. Butt

**Affiliations:** ^1^ Department of Haematology Aneurin Bevan University Health Board Newport UK; ^2^ Department of Haematology University College London Hospital NHS Foundation Trust London UK; ^3^ Department of Haematology Torbay and South Devon NHS Foundation Trust Torquay UK; ^4^ Department of Haematology Gloucestershire Hospitals NHS Trust Gloucester UK; ^5^ Department of Haematology Royal Cornwall Hospital NHS Trust Truro UK; ^6^ Department of Haematology Musgrove Park Hospital NHS Trust Taunton UK; ^7^ Department of Haematology Derriford Hospital NHS Trust Plymouth UK; ^8^ Department of Haematology Leicester Royal Infirmary NHS Trust Leicester UK; ^9^ Department of Haematology Beatson West of Scotland Cancer Centre Glasgow UK; ^10^ Department of Haematology University Hospital of Wales Cardiff UK; ^11^ Department of Haematology Royal Devon University Healthcare NHS Foundation Trust Exeter UK; ^12^ Department of Haematology the Newcastle Upon Tyne Hospitals NHS Foundation Trust Newcastle Upon Tyne UK; ^13^ Department of Haematology Clatterbridge Cancer Centre NHS Trust Liverpool UK

**Keywords:** essential, myelofibrosis, myeloproliferative disease, polycythaemia vera, thrombocythaemia

## Abstract

**Introduction:**

Myeloproliferative neoplasms (MPNs), such as polycythaemia vera (PV), essential thrombocythemia (ET) and myelofibrosis (MF), are primarily treated by managing blood counts to reduce the thrombotic risk using cytoreductive agents. Busulphan, an oral alkylating agent, has been historically used for MPN management due to its myelosuppressive effects, but concerns about its risk of leukaemic transformation have limited its use.

**Methods:**

This real‐world retrospective study evaluated the safety and efficacy of busulphan in 115 MPN patients across 13 UK hospitals. Responses in patients with ET and PV only were assessed using European LeukemiaNet (ELN) criteria.

**Results:**

With a median age of 78 years, the overall response rate was 78.1%, with 29% of PV and 18% of ET patients achieving complete responses. Dosing regimens were similarly distributed between repeated single doses of busulphan (31%), courses of treatment lasting 1–4 weeks (30%) and continuous therapy for more than 4 weeks (35%). No cases of disease progression to acute leukaemia or myelofibrosis were recorded during the median follow‐up of 23 months. Adverse events were infrequent, with fatigue and cytopaenia being the most common (4% each).

**Conclusion:**

Busulphan demonstrated a favourable safety profile and is a viable cytoreductive option, particularly for elderly patients who are intolerant to hydroxycarbamide.

**Trial Registration:**

The authors have confirmed clinical trial registration is not needed for this submission

## 1 Introduction

Myeloproliferative neoplasms (MPNs) are a diverse group of chronic myeloid malignancies that include polycythaemia vera (PV), essential thrombocythemia (ET) and myelofibrosis (MF). These disorders primarily affect older adults and are characterised by abnormal proliferation of blood cells, leading to a range of complications such as thrombosis, haemorrhage and potential transformation to acute myeloid leukaemia (AML). The treatment goals for MPNs are multifaceted, focusing on blood count control, reduction of thrombosis risk, symptom management, and in some patients, prevention of disease transformation to acute leukaemia and MF. Patients with MPNs who exhibit intolerance or resistance to standard therapies present a significant clinical challenge. In cases of intolerance, the therapeutic arsenal for these patients remains limited, highlighting the need for alternative treatments.

Busulphan, an alkylating agent, has been historically used in the treatment of MPNs due to its myelosuppressive properties. It is an attractive option since it is administered orally and is inexpensive. However, historical concerns were raised about its potential to cause leukaemic transformation [1, 2, 3, 4] and its implication as a risk factor for non‐melanoma skin cancer [5]. Despite these concerns, successful use of busulphan has been documented in previous retrospective studies, demonstrating efficacy and safety when used as a single agent in PV [6], ET [7] and more broadly in MPN patients [8, 9]. Importantly, there is evidence suggesting that busulphan therapy can reduce the *JAK2* V617F variable allele frequency (VAF) in patients with PV [10]. Current guidelines reflect this nuanced role of busulphan in MPN treatment. The British Society for Haematology recommends its use after third‐line treatment in patients with PV and limited life expectancy [11]. The European LeukemiaNet (ELN) guidelines suggest that intermittent doses of busulphan may be considered for very elderly patients with ET [12].

Despite these guidelines, there remains a lack of consensus and considerable variability in prescribing practices for busulphan. This study aims to evaluate the prescribing regimens, characteristics and outcomes of MPN patients treated with busulphan in a UK real‐world setting. We analysed data from multiple UK hospitals to understand the efficacy and safety of busulphan, focusing on outcomes based on varying dosing strategies.

## 2 Methods

We undertook a real‐world retrospective study across 13 MPN treatment centres in the United Kingdom. The study included patients diagnosed with MPNs, specifically those with PV, ET, MF and MPN, not otherwise specified (NOS). Patients with myelodysplastic/MPN were excluded.

Clinicians reviewed patient notes, and data were collected using a standardised pro forma. The variables recorded included baseline demographic and MPN characteristics, details of therapy including dosing and outcomes based on the ELN response criteria in patients with ET and PV as outlined by Barosi et al. [13]. The overall response rate (ORR) was defined as patients achieving a complete response (CR) or partial response (PR) as per the ELN criteria. Patients were administered with repeated courses of oral busulphan based on clinical response and tolerability. Continuous busulphan therapy was defined as busulphan administered for more than 4 weeks continuously.

Logistic regression was employed to evaluate the impact of potential prognostic characteristics on the ORR to busulphan, with results expressed as odds ratios (OR) and 95% confidence intervals (CI) in patients with ET and PV. The Cox proportional hazards model was used to assess the prognostic impact of baseline characteristics on overall survival, estimating hazard ratios (HR) and 95% CIs. The proportionality assumption for the Cox model was assessed using log–log plots. Significant variables identified from the univariate analysis were included in a multivariable Cox model to determine their independent prognostic significance.

All patient data were anonymised, and the study adhered to the ethical principles outlined in the Declaration of Helsinki and complied with the UK Data Protection Act (1998). In addition, the study received approval from the Aneurin Bevan University Health Board Research and Development department.

## 3 Results

Patient characteristics are summarised in Table 1. The study cohort comprised 115 patients, with a median age at diagnosis of 78 years (range: 36–96 years). Of the cohort, 51 (44%) were male and 64 (56%) were female. The median follow‐up period was 23 months (range: 0.25–218 months). The vast majority of patients were considered high risk, as defined by age over 60 years, a history of thrombosis and the presence of cardiovascular risk factors. Cardiovascular risk factors were present in 106 patients (92%) and included diabetes mellitus, hypertension, dyslipidaemia and previous arterial thrombosis. A history of thrombosis was recorded in 42 patients (22%), with arterial thrombosis in 25 patients (16%), venous thrombosis in 14 patients (12%) and both types in 3 patients (3%).

**TABLE 1:** Patient demographics and clinical phenotype.

Patient characteristics, *n* = 115	
Age at diagnosis, median (range), years	78 (36–96)
Sex	
Male	51 (44%)
Female	64 (56%)
Follow‐up, median (range), months	23 (0.25–218)
Mutational status	
*JAK2*	71 (62%)
*CALR*	15 (13%)
*MPL*	8 (7%)
Triple‐negative	4 (3%)
Unavailable	17 (15%)
Diagnosis	
Essential thrombocythemia	77 (67%)
Polycythaemia vera	28 (24%)
Myelofibrosis	4 (4%)
Myeloproliferative neoplasm, NOS	6 (5%)
Cardiovascular risk factor	
Present	106 (92%)
Dementia	
Present	9 (8%)
History of malignancy	
Total	18 (16%)
Non‐melanoma skin cancer (BCC, SCC)	9 (8%)
Other	9 (8%)
Melanoma	1 (1%)
Low grade lymphoma	2 (2%)
Breast cancer	4 (4%)
Prostate cancer	1 (1%)
Lung cancer	1 (1%)
History of thrombosis	
Total	42 (37%)
Arterial thrombosis	25 (22%)
Venous thrombosis	14 (12%)
Both	3 (3%)
Previous cytoreductive therapies	
Hydroxycarbamide	90 (78%)
Anagrelide	19 (16%)
Pegylated interferon	9 (8%)
P32	3 (3%)
Ruxolitinib	2 (2%)
Nil	15 (13%)
Line of therapy	
1st line	15 (13%)
2nd line	72 (63%)
3rd line	22 (19%)
4th line	6 (5%)

Abbreviations: BCC, basal cell carcinoma; NOS, not otherwise specified; P32, phosphorus‐32; SCC, squamous cell carcinoma.

Regarding mutational status, 71 patients (62%) harboured the *JAK2* V617F mutation. *CALR* mutations were present in 15 patients (13%), MPL mutations in 8 patients (7%) and 4 patients (3%) were classified as triple‐negative. Mutational status was unavailable for 17 patients (15%).

The majority of patients were diagnosed with ET (77 patients, 67%), followed by PV in 28 patients (24%), MF in 4 patients (4%) and MPN and NOS in 6 patients (5%).

Most patients (*n* = 90, 78%) had received hydroxycarbamide as their primary cytoreductive therapy prior to treatment with busulphan, highlighting its role most commonly beyond first‐line therapy. Other prior treatments included anagrelide (*n* = 19, 16%), pegylated interferon (*n* = 9, 8%), radioactive phosphorus (P32) (*n* = 3, 3%) and ruxolitinib (*n* = 2, 2%). A total of 15 patients (13%) had not received any prior cytoreductive therapy.

**FIGURE 1:**
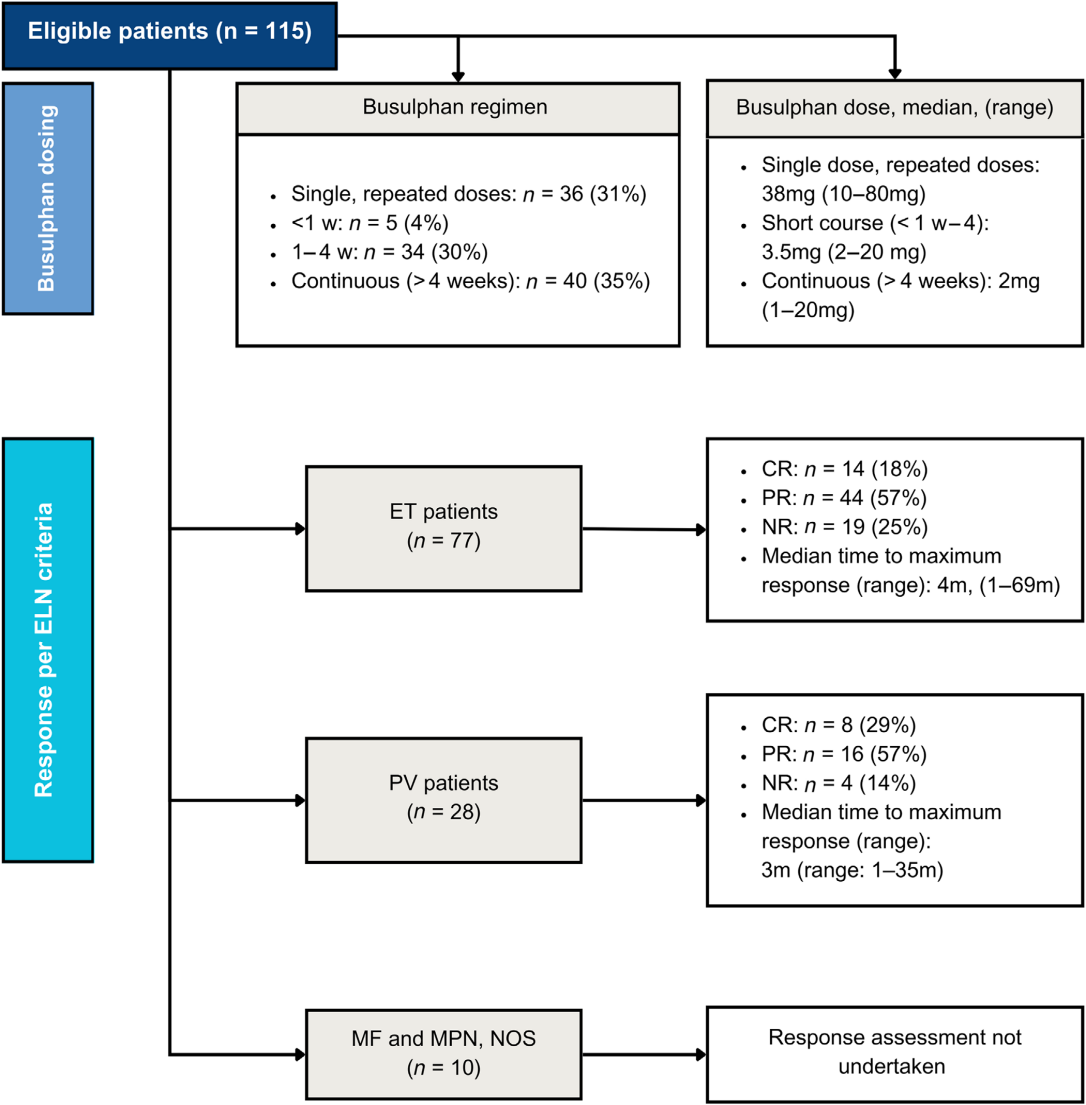
Consort diagram. CR, complete response; ELN, European LeukemiaNet; ET, essential thrombocythemia; m, months; MF, myelofibrosis; MPN, myeloproliferative neoplasm; NOS, not otherwise specified; NR, no response; PR, partial response; PV, polycythaemia vera; w, weeks.

The most common busulphan regimen was continuous therapy (> 4 weeks), administered to 40 patients (35%) (Figure 1). This was followed by repeated single doses in 36 patients (31%) and a 1–4‐week course in 34 patients (30%). A short course of less than 1 week was given to 5 patients (4%). The median busulphan dose for the repeated single‐dose regimen was 38 mg (range: 10–80 mg). For the short course (< 1–4 weeks), the median dose was 3.5 mg (range: 2–20 mg) and for the continuous regimen, the median dose was 2 mg (range: 1–20 mg).

Reflected in Figure 1, when examining treatment outcomes according to the ELN criteria, in patients with PV, 8 patients (29%) achieved a CR, 16 patients (57%) achieved a PR and 4 patients (14%) had no response (NR). Among patients with ET, 14 patients (18%) achieved a CR, 44 patients (57%) achieved a PR and 19 patients (25%) had NR. The time to maximum response was 3 months (range: 1–35 months) for patients with PV and 4 months (range: 1–69 months) for those with ET.

The median length of busulphan therapy, from initiation to last follow‐up or death, was 23 months (range: 0.25–218 months). During the follow‐up period, 25 patients (22%) died, though none of these deaths were attributed to busulphan therapy. Importantly, no cases of progression to AML or MF were documented, and there were no reports of secondary malignancies or thrombotic episodes. Among patients no longer on busulphan at last follow‐up, 17 patients (14%) were alive, had acceptable blood count control, and were not receiving any other cytoreductive therapy. Treatment cessation in these patients was deemed appropriate by the treating physician based on clinical judgment and individual patient factors.

Complications related to busulphan were noted in 16 patients (14%). These included nausea (3%), fatigue (4%), cytopaenia (4%), infection (2%) and worsening dementia (1%). Only 20 patients (17%) remained on busulphan at last follow‐up.

Multivariate logistic regression analysis did not identify any features that were independently associated with the ORR at the 5% significance level. However, male sex exhibited a trend towards higher ORR (OR: 2.47, 95% CI: 0.88–6.94, *p* = 0.10) and age at diagnosis demonstrated a potential association with response (OR: 1.04 per year, 95% CI: 0.99–1.09, *p* = 0.086), though neither reached statistical significance. Other factors, such as having a primary diagnosis of PV (OR: 2.39, 95% CI: 0.63–9.10, *p* = 0.20) and receiving a busulphan regimen lasting more than 4 weeks (OR: 1.84, 95% CI: 0.49–6.92, *p* = 0.36), were not significantly associated with ORR.

## 4 Discussion

This study reports the largest modern‐era cohort of MPN patients treated with busulphan, providing valuable evidence on its utility, especially in patients previously treated with hydroxycarbamide and high‐risk features. Busulphan was shown to be safe and well‐tolerated, with a manageable side effect profile. The drug demonstrated efficacy in rapidly controlling blood counts, with an ORR of 78.1%, and no significant differences in outcomes were observed across different dosing patterns, including single‐dose regimens, short courses and continuous dosing.

Reassuringly, there was no evidence of disease transformation to MF or AML during the follow‐up period, which has historically been a concern with busulphan use. These findings support the ongoing use of busulphan as a viable cytoreductive option in older patients, particularly those who may not tolerate more commonly used agents like anagrelide and interferon. Importantly, while we did not assess *JAK2* V617F VAF in this study, the effect of busulphan on *JAK2* VAF represents an important exploratory research question.

The retrospective nature of the study inherently introduces biases related to incomplete data collection and variability in treatment administration. The lack of detailed dosing information, especially the number of cycles used, also limits our ability to make definitive conclusions about optimal busulphan regimens. In addition, the relatively short median follow‐up period may have underestimated long‐term complications or disease progression, such as the potential development of MF or AML, though no cases were documented in this cohort.

Our data also highlight a challenge in applying the ELN criteria [13] in real‐world settings. The ELN criteria, designed for clinical trials, may be too stringent for older patients with multiple comorbidities, cytopaenias or cognitive impairment. In practice, clinicians may need to adopt more flexible thresholds for treatment response, balancing efficacy with patient‐specific factors.

Based on these findings, we propose that busulphan should be considered an acceptable alternative to standard cytoreductive therapies in patients with a life expectancy of less than 10 years. It can be administered as a short course or even as a single dose in cases where regular dosing is difficult. Single‐dose regimens, in particular, may be especially useful in elderly patients with compliance issues. Close and continuous monitoring for the development of secondary malignancies, especially non‐melanoma skin cancers, is warranted and should not be omitted due to the short duration of most busulphan therapy regimens.

Given the lack of prospective studies on the use of busulphan, our study contributes valuable real‐world data on the safety and efficacy of busulphan which clinicians should find useful in managing this challenging cohort.

## Author Contributions

A.M. designed the study, performed the analysis and wrote the first draft of the manuscript. A.R. wrote the abstract. A.M., P.R., J.S., E.M., R.W., D.M., N.I., G.N., M.G., I.M., P.G., G.K., S.B., J.C., A.M., R.F. and N.B. contributed patients. All authors reviewed and approved the final draft of the manuscript.

## Conflicts of Interest

Ali Mahdi: Advisory board for GSK and Novartis. Speaker fees from Incyte, Novartis and Pfizer. Andrew McGregor: Advisory board for Novartis. Speaker fees from GSK and Novartis. Rebecca Frewin: Advisory board for GSK. Conference sponsorship from Novartis. The other authors declare no conflicts of interest.

## Data Availability

The authors have nothing to report.
